# 1013. Case Study. Fish Skin Grafts for Partial-thickness Electrical Burns: A Mini Case Series of Successful Outcomes

**DOI:** 10.1093/jbcr/irag033.201

**Published:** 2026-04-06

**Authors:** Anna M Malysz Oyola, Mia Pepi, David Parizh

**Affiliations:** Nassau County Firefighters Burn Center at Nassau University Medical Center, East Meadow, NY; Nassau County Firefighters Burn Center at Nassau University Medical Center, East Meadow, NY; Nassau County Firefighters Burn Center at Nassau University Medical Center, Garden City, NY

## Abstract

**Patient Presentation (age range, injury details, relevant history):**

A 27-year-old and 36-year-old healthy Hispanic male with Fitzpatrick type IV skin sustained 1% electrical burns to their left (non-dominant) hands during a construction accident. The 27-year-old had superficial and deep second-degree burns to dorsal digits 3-5 (including PIP and DIP joints), with near circumferential wrist involvement. The 36-year-old had superficial second-degree burns to all dorsal digits (including MCP, PIP, and DIP joints) and volar wrist involvement.

**Clinical Challenges:**

Deep partial thickness burns spanning multiple joints. The goal was to reduce scarring and optimize hand function, focusing on a quick return to work, effective pain management, and brief hospitalization.

**Management Approach:**

Both patients underwent 5 days of local wound care followed by tangential excision and piscine xenograft coverage. Wound care included twice daily cleansing, Silvadene application, gauze wrap, elastic compression and elevation. Occupational therapy was consulted. Tangential excision was performed with a Goulian blade, followed by the application of piscine xenografts fixed with staples. Sterile dressings included antibiotic ointment, gauze wrap, and an elastic compression bandage. Both patients were discharged with one-week follow-up for dressing changes.

**Outcomes:**

A mini case series with terrific cosmetic and functional outcomes while providing prompt coverage of a raw wound, thus minimizing pain.

Piscine xenograft was pursued in lieu of autograft due to the superficial partial thickness depth. The piscine dermal matrix offered the additional benefits of modulating inflammation, promoting neovascularization and reinforcing the dermal construct with natural collagen, elastin, glycosaminoglycans and extracellular membrane components resulting in a more pliable neodermis.

At 1 week post-op, full graft incorporation was noted in deeper areas, while more superficial injuries exhibited partial graft loss as dry, scaly tissue. Neither patient required narcotics postoperatively. Both had some restricted grip formation, more pronounced in the younger male. They were provided wound care, hand exercises, and outpatient occupational therapy prescriptions.

At 2 weeks post-op, both patients had full epithelialization, no hypertrophy, and improved range of motion. Wound care transitioned to moisturizing, and they were provided compression gloves and sun exposure precautions. They returned to work at this time without functional deficits.

At 7 months post-op, both patients had excellent cosmetic outcomes and regained native functionality, with no hypertrophy or contracture. The patients' lack of co-morbidities and good nutritional status likely aided healing.

**Lessons Learned:**

In appropriate cases, piscine xenografts can achieve timely wound healing with optimal tissue pliability and cosmesis without the need for autograft, thus reducing procedural time, minimizing pain, and avoiding donor site morbidity.

**Applicability to Practice**:

